# Targeting Lymphatics for Nanoparticle Drug Delivery

**DOI:** 10.3389/fphar.2022.887402

**Published:** 2022-06-03

**Authors:** Jacob McCright, Ritika Naiknavare, Jenny Yarmovsky, Katharina Maisel

**Affiliations:** Department of Bioengineering, University of Maryland College Park, College Park, MD, United States

**Keywords:** immunotherapy, lymph node, nanomaterial, lymphatic endothelial cells, barrier

## Abstract

The lymphatics transport material from peripheral tissues to lymph nodes, where immune responses are formed, before being transported into systemic circulation. With key roles in transport and fluid homeostasis, lymphatic dysregulation is linked to diseases, including lymphedema. Fluid within the interstitium passes into initial lymphatic vessels where a valve system prevents fluid backflow. Additionally, lymphatic endothelial cells produce key chemokines, such as CCL21, that direct the migration of dendritic cells and lymphocytes. As a result, lymphatics are an attractive delivery route for transporting immune modulatory treatments to lymph nodes where immunotherapies are potentiated in addition to being an alternative method of reaching systemic circulation. In this review, we discuss the physiology of lymphatic vessels and mechanisms used in the transport of materials from peripheral tissues to lymph nodes. We then summarize nanomaterial-based strategies to take advantage of lymphatic transport functions for delivering therapeutics to lymph nodes or systemic circulation. We also describe opportunities for targeting lymphatic endothelial cells to modulate transport and immune functions.

## Introduction

Lymphatic vessels exist throughout the body and are mainly appreciated for transporting extracellular fluid and solutes including antigens, cells, extracellular vesicles, and particulates to draining lymph nodes and eventually into systemic circulation. Lymphatic vessels have traditionally been thought of as passive transporters; however, recent studies have found that lymphatic vessels actively transport materials and also play a key role in modulating immunity. The natural transport functions of lymphatic vessels have been used to deliver immunomodulatory therapies to lymph nodes to shape immune responses. This can be achieved using nanomaterial formulations that preferentially drain into lymphatic vessels. In this review we provide an overview of lymphatic vessel function and physiology, highlight therapeutic formulations developed to target lymphatic vessels, and thus the downstream lymph nodes to enhance immunotherapies, and explore how directly targeting lymphatic immune functions modulate immunity.

## Lymphatic Vessel Physiology

### Initial and Collecting Lymphatics

**FIGURE 1:**
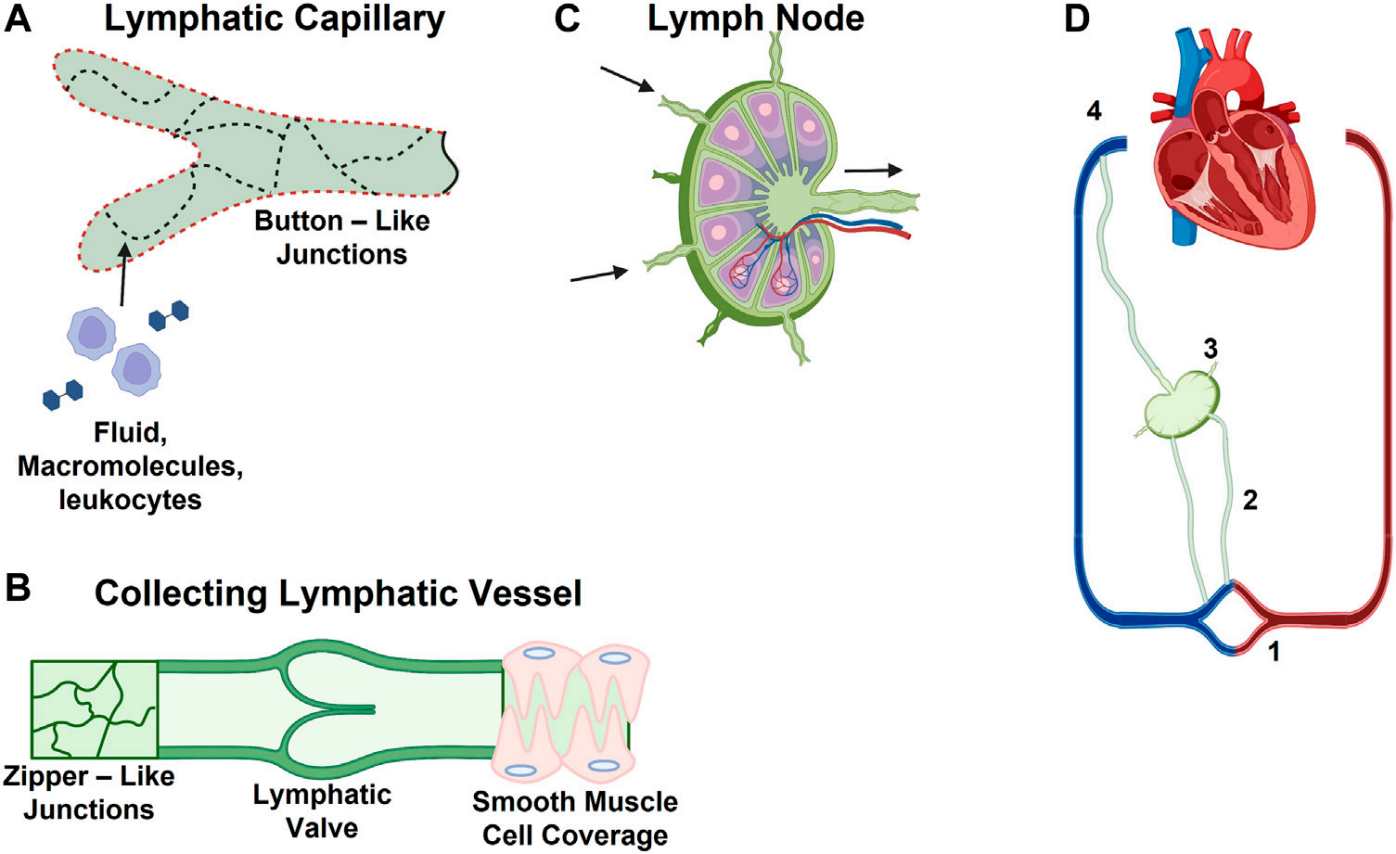
Schematic of Lymphatic Vessel Transport Properties. **(A)** Discontinuous basement membrane (red dashed line) and button junctions (dotted lines) allow for lymphatic capillaries to absorb interstitial solutes, macromolecules, and immune cells. **(B)** Collecting vessels contain zipper-like junctions (continuous lines) and unidirectional valves. **(C)** Schematic of lymph node with multiple afferent and a single efferent vessel. **(D)** Lymphatic vascular system consists of (1) lymphatic capillaries, (2) collecting lymphatic vessels, (3) lymph nodes, and (4) the thoracic duct and right lymphatic trunk.

Initial lymphatics, also known as lymphatic capillaries, are the entry point for lymph and the initial pathway into the lymphatics system. Made up of vessels ranging from 10 to 60 μm, initial lymphatics form a network in interstitial spaces of tissues (Figure 1A (Aspelund et al., 2016)) (Scallan et al., 2010). The lymphatic capillary walls are made up of single-layered cells that overlap in some areas, giving the appearance of one-way flaps (Baluk et al., 2007), or microvalves (Trzewik et al., 2001). Although initial lymphatics are blind-ended, it is postulated that the microvalves embedded within their capillary walls allow for permeation of larger molecules, such as proteins and particulates (Ikomi et al., 1996). The microvalves of the capillaries connect directly to surrounding tissues via thin, elastic fibers. These fibers only connect to the external wall of the initial lymphatics, leaving the internal surface unattached and flexible (Scallan et al., 2010). Initial lymphatics transport materials toward collecting lymphatics or collecting vessels. Collecting lymphatics have been classified as two different types: afferent vessels that transport lymph from peripheral tissue to the lymph nodes, and efferent vessels that transport lymph away from the lymph node to systemic circulation (Figures 1B,C) (Schulte-Merker et al., 2011). Typically, afferent lymph consists of lymphocytes, and antigen-presenting dendritic cells (Pflicke and Sixt, 2009; Haig et al., 1999). Dendritic cells enter the afferent vessels by leaking through peripheral tissue and blood capillaries into the interstitial space. From there, they can migrate into initial lymphatics. During inflammation, dendritic cells can be recruited to the lymphatic vessels by lymphatic endothelial cells (LECs) through increased production of chemokines and LEC surface markers including CCL21, and CCL22 (Lucas and Tamburini, 2019; Vigl et al., 2011). These chemokines are ligands for CCR7, which recruits T and dendritic cells to the lymphoid tissue. They are also important in thymocyte development and secondary lymphoid organogenesis (Comerford et al., 2013). The resulting increase of dendritic cells within the lymph nodes promotes lymph node expansion and additional secretion of chemokines by other stromal cells recruits lymphocytes to the lymph nodes (Lucas and Tamburini, 2019). Efferent vessels transport lymph from the lymph nodes to the large vessels, which eventually return the lymph back into blood circulation. Efferent lymphatic vessels carry lymphocytes, in particular a high proportion of CD4^+^ T cells and B cells, compared to CD8^+^ and γδ T cells (Haig et al., 1999). Efferent lymphatic vessels converge and carry material towards the thoracic duct and the right lymphatic trunk, then drain material into venous circulation via four lymphovenous valves where the subclavian and jugular veins meet (Figure 1D) (Aspelund et al., 2016; Geng et al., 2016).

### Barrier Functions of Lymphatic Vessels

Lymphatic vessels form a barrier between the interstitial space of tissues and lymph within the lumen of the vessels. Lymphatics are semipermeable and only allow unidirectional flow. Initial and collecting lymphatics have different functions, and the unique structures of the vessels enable those functions. Initial lymphatic vessels are composed of LECs surrounded by an incomplete basement membrane (Breslin et al., 2018). Initial vessels facilitate the formation of lymph, so they require high permeability to allow for the transport of fluid, macromolecules, lipids, and immune cells away from the interstitial space of tissues. Transport into lymphatics occurs when local fluid accumulation and tissue stress result in higher pressure in the interstitial fluid than the lumen of lymphatic vessels. When this occurs, lymph is transported into vessels through paracellular and transcellular routes (Triacca et al., 2017). Conversely, if pressure is higher inside the lymphatic vessels, endothelial cells adhere more closely, preventing lymph from flowing back into interstitial fluid. Collecting vessels are larger vessels that transport lymph collected form several initial vessels to the lymph node, requiring lower permeability and a stronger barrier to prevent lymph leakage. Collecting vessels are surrounded by a continuous basement membrane and perivascular cells. Shear stress within collecting vessels causes a tightening of the lymphatic endothelial barrier through cell junction stabilization, and thus enhances barrier functions (Sabine et al., 2015; Breslin et al., 2018). This is likely part of a mechanism to prevent excessive leakage and optimize lymph flow. While shear stress results in increased barrier function, inflammation generally has the opposite effect. During inflammation, upregulation of inflammatory cytokines can lead to increased lymphatic endothelial cell permeability within initial lymphatics (Schwager and Detmar, 2019). This occurs through disruption of cell-cell junctions and contraction of the actin/myosin cytoskeleton within LECs (Cromer et al., 2014). Impaired lymphatic endothelial barrier function can result in several pathological conditions. For instance, edema develops if gaps between endothelial cells are too large (Scallan et al., 2010). Furthermore, in lymphedema, lymphatic vessels experience high levels of shear stress and stretch and adapt to these conditions by increasing transport (Rahbar et al., 2014).

### Cell-Cell Junctions

The junctions of lymphatic vessels are largely composed of the adherens junction proteins VE-cadherin, β-catenin and p120-catenin, and the tight junction proteins occludin, claudin-5 and zonulin-1 (ZO-1), junctional adhesion molecule-A (JAM-A), and endothelial cell-selective adhesion molecule (ESAM) (Zhang et al., 2020). Despite the similarities in composition, junctions in initial and collecting lymphatic vessels differ greatly in morphology and function (Dejana et al., 2009). Initial lymphatic vessels have discontinuous junctions, also referred to as button-like. Button junctions are characterized by discontinuous segments of VE-cadherin at the border of endothelial cells (Baluk et al., 2007). They are formed by the overlap of the membranes of endothelial cells, which create flap-like mini-valves (Trzewik et al., 2001). In normal conditions these flaps are closed and prevent flow from the lumen into the interstitial space. The tips of the flaps have filaments that attach the endothelial cells to the extracellular matrix. In the presence of a pressure gradient from the interstitial space to the lumen, these filaments pull on the flaps, creating a separation between the cells that allows fluids and solutes to enter lymphatic vessels (Schwartz et al., 1999). An advantage of this structure is that drainage into lymphatics can occur without the dissolution of endothelial cell junctions, maintaining vessel integrity (Pflicke and Sixt, 2009). Since collecting lymphatic vessels do not exchange materials with surrounding tissue, they have different types of junctions called zipper-like junctions, which provide a strong barrier between the vessel and its surroundings through continuous localization of VE-cadherin at the lymphatic endothelial cell borders (Yao et al., 2012). During inflammation, the alteration of both button- and zipper-like junctions can increase lymphatic permeability (Kakei et al., 2014).

### Fluid Homeostasis

Regulating fluid balance is crucial in bodily function (Swartz, 2001). Most plasma produced by the body is returned to the bloodstream by veins. However, the excess fluid that is not returned, or leaks through the blood capillaries, remains behind and accumulates in the interstitial spaces between tissues. One of the main roles of the lymphatic system is to maintain balance by absorbing that excess fluid, known as lymph, and returning it back to systemic circulation (Miteva et al., 2010). Fluid moves unilaterally through the lymphatic system, first absorbed by the initial lymphatics, transported via collecting lymphatics across lymph nodes, and eventually enters either the right lymphatic duct or the thoracic duct, near the base of the neck (Hematti and Mehran, 2011). Without proper lymphatic drainage, the buildup of fluid in the interstitial spaces can potentially cause life-threatening conditions (Casley-Smith, 1985). One such example is lymphedema, which occurs when excess interstitial fluid builds up in tissues, leading to distension, inflammation, swelling of the limbs, head, and neck, and an increase in fatty tissue significantly affecting quality of life (Velanovich and Szymanski, 1999; Rockson, 2001; Gupta and Moore, 2018).

## Targeting Lymphatics for Drug Delivery

Immunotherapies, including vaccines, have been extremely promising for curing diseases including cancer, chronic inflammation, and transplantation. Delivery of these treatments to the draining lymph nodes, where adaptive immunity is formed, amplifies their efficacy, thus potentially improving their clinical outcomes. One strategy to target lymph nodes indirectly from peripheral tissues via, e.g., intramuscular or subcutaneous injection, is to use lymphatic transport functions. Nanoparticles are an attractive vehicle to transport vaccine and vaccine adjuvant to lymph nodes through lymphatic vessels. Nanoparticles can be engineered to facilitate the controlled release of immunotherapies, and can limit negative immunogenic and toxic side effects through the encapsulation of delivered components and targeted delivery to tissues of interest (Reddy et al., 2007; Gutjahr et al., 2016; Chen et al., 2020). Research from the last decade has identified that nanomaterials between 10—250 nm in diameter (Kobayashi et al., 2006), and up to 1 micron in some studies, can also be taken up by LECs through transcellular and paracellular mechanisms (Srinivasan et al., 2016; McCright et al., 2022). In the gastrointestinal tract, lymphatics also play a special role in lipid absorption: lipids are taken up by lymphatic vessels *via* chylomicrons, small lipid vesicles into which dietary lipids are packaged by enterocytes. This can be taken advantage of for drug delivery, as targeting gastrointestinal lymphatics circumvents therapeutics from being digested via hepatic first pass metabolism. In this section we describe some of the various nanomaterial-based strategies for targeting lymph nodes via lymphatic vessels, as well as lipid-based strategies to target gastrointestinal lymphatics. These approaches are summarized in Table 1.

**TABLE 1:** Summary of nanoparticle formulations used for lymphatic delivery.

Nanoparticle formulation	Advantages	Disadvantages	Lymphatic application	Sources
**Polymeric Nanoparticles**	Can be biodegradable. Flexible formulation allows for precise tissue and cell targeting and controlled release. High stability	Difficult to scale production. Possible toxicity of polymer	PEGylation allows for improved entry into lymphatics and delivery to lymph nodes. Formulations have been engineered to release cargo in lymph nodes	[36, 38, 42–44, 49, 65]
**Liposomes and Micelles**	Easy formulation that allows for some surface modification. Nontoxic and biodegradable	Low solubility Short circulation times	Liposomes can effectively encapsulate insoluble drugs	[37, 38, 45, 47, 55]
**Dendrimers**	Highly tunable chemical and physical properties. Covalent association of drugs	High cost of formulation. Often outside the size range for effective lymphatic transport (10—250 nm)	Serve as solubility enhancers for drugs	[39, 41]
**Solid Lipid Nanoparticles**	Biocompatible Flexible formulation can aid in tissue specificity	Difficulty scaling production. Poor solubility	Incorporation of pro-drugs can impart lymph node and lymphatic vessel delivery after oral administration	[54, 56, 58, 60–64]

Bold values within the table are types of nanoparticles, with their features described within the row.

### Nanomaterial-Based Lymphatic Targeting

Nanoparticle size was one of the first parameters investigated with respect to optimizing lymphatic drug delivery. A study from the Swartz lab showed that fluorescent 20—100 nm, PEG-stabilized poly(propylene sulfide) nanoparticles accumulated within LNs after intradermal tail injection. Importantly, they were able to visualize the lymphatic network of the mice using fluorescent nanoparticles (Reddy et al., 2007). This visualization was also dependent on nanoparticle size, smaller 20 nm nanoparticles were present in higher quantities within lymphatic vessels compared to larger 100 nm nanoparticles. When lymph nodes were recovered, the 20—45 nm nanoparticles were found co-localized with MHCII^+^ antigen-presenting cells. Additionally, when these nanoparticles were conjugated to model antigen ovalbumin, they found that the humoral and cellular immune response in mice was generated in a size-dependent manner. Nakamura et al. also observed a similar phenomenon when subcutaneously injecting pH-sensitive lipid nanoparticles in inguinal regions of mice. They found that the 30 nm formulations were transported into lymph nodes and taken up by CD8^+^ dendritic cells more effectively than 100 nm and 200 nm formulations of the solid lipid nanoparticles (Nakamura et al., 2020). Recent work by our group has also found that polyethylene glycol (PEG)-coated 40 nm nanoparticles were able to cross lymphatic barriers more efficiently than 100 nm PEG-coated nanoparticles (McCright et al., 2020).

The size range required for targeting lymphatic vessels is now well-established, so many studies have turned to understanding the surface chemistry requirements necessary for lymphatic targeting. Varypataki compared anionic poly(lactic-co-glycolic) (PLGA) nanoparticles and cationic liposomes (Varypataki et al., 2016). They found that the 180 nm cationic liposome vaccine formulations reached lymph nodes and were more effective in elucidating *in-vivo* immune response after subcutaneous injection compared to the 350 nm PLGA nanoparticles. This improved potency and delivery of cationic liposome vaccines is likely to be at least in part due to the smaller size of the liposome formulations compared to the PLGA formulations. A study from Nishimoto et al. examined the effect that charge had on dendrimer delivery to lymph nodes following intradermal injection (Nishimoto et al., 2020). They found that anionic dendrimers accumulated within lymph nodes, with phosphate-terminal dendrimers recognized by the macrophages, dendritic cells, and B cells in the lymph node, whereas other anionic dendrimers were not. However, all formulated dendrimers were <10 nm in diameter, suggesting that they are outside the range of sizes for optimum lymphatic transport (Rohner and Thomas, 2017). Research from Kaminskas et al. demonstrated that PEGylation of poly-l-lysine dendrimers enhanced their transport to the LNs after subcutaneous injection in murine models (Kaminskas et al., 2009). However, similar to the study by Varypataki, there were significant size differences in the compared formulations. Notably, non-PEGylated dendrimers were under 10 nm in size, falling outside of the range identified for optimal lymphatic transport (Rohner and Thomas, 2017).

Early studies from Moghimi et al. demonstrated that coating nanoparticles with polyethylene glycol (PEG)-polypropylene oxide copolymers can enhance LN accumulation of nanoparticles after intradermal administration in mice (Moghimi et al., 1994). De Koker et al. also demonstrated that modifying nanoparticle surface chemistry can be engineered to leverage lymphatic transport (De Koker et al., 2016). They were able to demonstrate that coating 200 nm mesoporous silica nanoparticles with PEG improved LN accumulation of nanoparticles after 12 and 48 h, compared to uncoated 200 nm silica nanoparticles. They were also able to demonstrate that PEG-coated nanoparticles were more effective at priming antigen-specific T cells (De Koker et al., 2016). Rao et al. also found that 50, 100, and 200 nm PEG-coated nanoparticles accumulated more in the LNs after subcutaneous injection compared to uncoated PLGA nanoparticles of the same size, suggesting that hydrophilicity is crucial to maximize lymphatic transport of nanoparticles (Rao et al., 2010). However, in this study, the surface potential of PEGylated nanoparticles was only -36.1 ± 14.6 mV, suggesting that the PEG coating was not very dense. Zhang et al. also demonstrated that the addition of PEG can improve liposome delivery to lymphatic vessels and lymph nodes (Zhuang et al., 2012). They demonstrated that the addition of 2 kDa PEG to the surface of 250 nm DOTAP liposomes expedited lymphatic transport and improved LN retention compared to DOTAP liposomes without PEG. While these findings have been critical in identifying nanoparticle surface chemistry required for indirect lymph node delivery, whether nanoparticles are being transported through lymphatic vessels or reaching lymph nodes through capture and transport with immune cells needs to be addressed in future studies (Chen et al., 2020). Recent work from our group has closely examined the role that surface chemistry plays in lymphatic permeability and transport. Using model 40 and 100 nm polystyrene-core nanoparticles, we found that the addition of PEG improved lymphatic permeability within a transwell lymphatic model compared to uncoated polystyrene nanoparticles. Additionally, we observed that increasing PEG density on the surface of the nanoparticles optimized transport across lymphatic endothelial cell barriers. Indeed, densely PEGylated nanoparticles were found to accumulate in draining lymph nodes within 4 h after intradermal injection. In addition to identifying optimum surface characteristics for lymphatic delivery, we also provided some of the first insight on the transport mechanisms involved in the transport of nanoparticles across lymphatic barriers. We found that both paracellular and transcellular transport mechanisms were key in crossing lymphatic barriers, with LECs relying on clathrin-mediated endocytosis to mediate transport of PEGylated nanoparticles (McCright et al., 2020).

Other studies have examined how nanoparticle formulation influence where they accumulate within targeted LNs. A study from Zeng et al. demonstrated that positively charged 30 nm polyethyleneimine-stearic acid micelles preferentially accumulated in draining LNs compared to free antigen (Zeng et al., 2015). They found that the nanoparticles were in the medulla and paracortex of lymph nodes, where T cells reside, following subcutaneous injection. Additionally, immunofluorescence imaging of LN sections showed significant nanoparticle accumulation around the border of the LN, within the subcapsular sinus. This result is consistent with reports that the conduit system within LNs is incapable of transporting material greater than 70 kDa from peripheral regions of the LN to central regions (Roozendaal et al., Kraal). In addition to demonstrating nanoparticles were able to effectively reach LNs, they also showed that the nanoparticle-based cancer vaccine was more effective in inhibiting tumor growth compared to free antigen.

Work from the Thomas group has provided some of the most cutting-edge technologies for delivering nanoparticles to lymph nodes via lymphatic vessels. Recently Schudel et al. generated a two-stage system inspired by the way particulate antigen is processed by the immune system. An outer stage comprised of F127 pluronic-containing PEG utilizes lymphatic transport to reach draining lymph nodes while protecting the cargo from systemic exposure. The second stage utilizes OND-thiol linkage chemistry programmed to release small molecule cargo when exposed to lymph. They were able to demonstrate that this technology allows for precise delivery of small molecules to lymph node—resident dendritic cells as well as controlled release through tuning of the degradation kinetics of the thiol bond (Schudel et al., 2020). Additional studies from the group can be highlighted in a recent review from the group (Kim et al., 2021).

### Lipid-Based Targeting of Lymphatics

Lymphatics are the key conduit for transporting dietary lipids from the gut into systemic circulation. Lipids typically enter enterocytes within the gastrointestinal tract through a receptor-mediated endocytosis or micropinocytosis (Cooper, 1997; Chaudhary et al., 2014). In the gastrointestinal epithelium, they are packaged into chylomicrons and exocytosed from the basolateral side of the enterocyte cell into the lamina propria, where chylomicrons effectively enter initial lymphatic vessels. Chylomicrons eventually enter systemic circulation through the thoracic duct that drains into the subclavian vein. The chylomicron processing pathway provides multiple unique opportunities for engineering drug delivery strategies as it signifies a route to reach lymph nodes to potentiate immunotherapies, as well as a way to achieve systemic delivery through an orally administered drug while simultaneously bypassing hepatic first pass metabolism.

To best utilize the chylomicron processing pathway for lymphatic delivery, therapeutics can be formulated as a prodrug consisting of a cleavable lipid component that triggers this innate transport pathway. Several groups have published extensively on how triglyceride-, and other fatty acid-, mimicking lipid formulations are able to improve the delivery of a variety of drugs to lymphatic vessels and gut-draining lymph nodes after oral administration (Trevaskis et al., 2015a; Lee et al., 2019; Kochappan et al., 2021; Lee et al., 2021). Indeed, the researchers’ development of a lymphatic-cannulation technique has allowed them to directly observe the transport of lymph from the small intestine through lymphatic vessels and lymph nodes that converge at the superior mesenteric lymph duct (Trevaskis et al., 2015b). This technique was also applied to examine the fate of subcutaneously administered, high-density lipoprotein nanoparticles (HDLs), finding that HDLs preferentially drained via lymphatic vessels, as opposed to blood vessels, and that LN retention of HDL was positively correlated to increasing the negative charge of HDL (Gracia et al., 2020). For more detailed descriptions of such prodrug formulations, we direct readers to this recent review (Dahan et al., 2014).

More recently, several groups have used the chylomicron pathway to delivery particulate cargo to intestinal lymphatics. Mao et al. generated 100–120 nm mesoporous silica nanoparticles that were coated with diglycerides to trigger chylomicron processing (Mao et al., 2019). They demonstrated that resident lipases within the gastrointestinal lumen cleaved the fats on the surface of the nanoparticle, prompting uptake and processing into chylomicrons, and further transport into lymphatic vessels. To confirm that the nanoparticles were being processed as chylomicrons, they probed the intracellular pathways using a combination of transport inhibitors and confirmed how chylomicron processing was necessary for transcellular transport of the nanoparticle. They also confirmed that these chylomicron-like nanoparticles were transported to the lymph nodes via lymphatic transport.

The Bae lab has used a similar strategy of promoting epithelial uptake of nanoparticles via bile-acid transporter-mediated cellular uptake, followed by chylomicron formation and transport into intestinal lymphatics. The group showed that 100 nm cationic solid lipid nanoparticles and carboxylate polystyrene nanoparticles coated with bile acids displayed significantly enhanced average oral bioavailability (47%) with sustained absorption in rats compared to uncoated nanoparticles (Kim et al., 2018; Kim et al., 2019; Suzuki et al., 2019). Baek et al. also used a similar strategy to delivery curcumin to the lymphatics (Baek and Cho, 2017). Solid lipid nanoparticles ranging from 150—250 nm were loaded with curcumin and administered orally. To prevent encapsulated drug release due to low pH conditions of the stomach, they coated their nanoparticle with chitosan, which also improved nanoparticle uptake into enterocytes. They found that lymphatic uptake and oral bioavailability of chitosan-coated solid lipid nanoparticles was 6.3-fold and 9.5-fold higher than that of curcumin solution, respectively.

A study by Lee et al. also examined how the addition of prodrug on the surface of their orally administered nanoparticle formulation could promote delivery to lymphatic vessels (Lee et al., 2021). Using a model lipophilic drug, Orilstat, they formulated emulsions containing medium-chain fatty acids, long-chain fatty acids, or long-chain triglycerides. Orilstat was found in the highest concentration in lymphatic vessels when coated with the long-chain fatty acids compared to the short-chain fatty acids and triglycerides. Drug concentration in the lymph peaked 2–3 h after oral administration of the drug. Increasing the presence of fatty acids on the surface of the emulsion was also found to improve nanoparticle transport across intestinal epithelial barriers and into lymphatic vessels.

Recently, Kochappan et al. formulated mycophenolic acid (MPA) to be attached to triglycerides. They hypothesized that the attached fatty acid would help the immunomodulatory drug reach lymphatic vessels and lymph nodes through the chylomicron processing pathway (Kochappan et al., 2021). Intraduodenal administration of the MPA-fatty acid conjugate improved drug concentration within lymph compared to MPA and MPA co-delivered (not conjugated). When examining MPA concentration within the lymph nodes, they found that the MPA-fatty acid conjugate resulted in a 20-fold higher concentration compared to MPA delivered alone.

## Lymphatic Endothelial Cells as Therapeutic Targets

In addition to transport and fluid regulation functions, the lymphatic endothelium plays a key role in immunity through interactions with immune cells. LECs are located throughout the entire body and produce key chemokines and adhesion molecules that help lymphatic vessels influence immunity.

### LEC-Mediated Immunity

Lymphatics are the highway for immune cells that enter peripheral lymphatic vessels within tissues and migrate to the local lymph nodes. Lymphatic vessels are key for the migration of dendritic cells, neutrophils, monocytes, and lymphocytes, including B and T cells, many of which migrate via the CCL21-CCR7 axis (Nykänen et al., 2010; Kriehuber et al., 2001). CCR7 is key in not only facilitating immune cell migration to the lymph nodes, but in directing dendritic cells to other tissues including the lamina propria, lungs, and skin. CCR7 has been shown to regulate dendritic cell association with collecting lymphatic vessels. Dendritic cell association with these collecting lymphatic vessels results in increased vessel permeability (Ivanov et al., 2016; Hampton and Chtanova, 2019). Additionally, dendritic cells preferentially enter lymphatics at sites where the CCL21 gradient is highest, suggesting that CCL21 directly regulates entry into lymphatics, a finding that is supported by the fact that intravital microscopy has revealed that CCL21 gradients also enhance dendritic cell migration within lymphatic vessels(69). CCL21 secreted by LECs also regulates the homing of naïve T cells from distant peripheral tissues to lymph nodes (Sallusto et al., 1999; Farnsworth et al., 2019). Additionally, CCR7 is a key discriminatory marker between central and effector memory T cells, with CCR7+ memory cells home towards lymph nodes and effectively stimulate dendritic cells (Sallusto et al., 1999).

While researchers have focused on lymphocyte and antigen presentation cell migration via lymphatics, more recent data demonstrates that neutrophils, one of the first immune cells to be recruited to a site of infection or injury, can also enter lymphatic vessels and migrate to the lymph nodes from these sites of inflammation (Gorlino et al., 2014; Tecchio et al., 2014; Rigby et al., 2015). The significance of this migration is not fully understood, it likely plays a role in preventing systemic pathogen spread (Kastenmüller et al., 2012; Takeda et al., 2019). Studies have indicated that there is a correlation between inflammation and neutrophil concentration in afferent lymph. Further discussion on the interplay between migratory immune cells and lymphatic vessels can be found here (Hampton and Chtanova, 2019).

In addition to regulating dendritic migration through the CCL21-CCR7 axis, LECs can interact directly with dendritic cells, which can impair dendritic cell maturation. Researchers have found that LECs were able to prevent dendritic cell maturation through ICAM-1/Mac-1 contact-dependent interactions, as well as through anti-inflammatory prostacyclin synthesis and TGF-β secretion (Garnier et al., 2019). Research has also shown that LECs have a key part in regulating T cell activation through the secretion of nitric oxide within the lymph nodes in the presence of inflammation (Lukacs-Kornek et al., 2011). One of the more interesting methods in which LECs can modulate immune responses is through the transfer of antigen to professional antigen-presenting cells, CD4^+^ cells, and CD8^+^ cells (Tamburini et al., 2014). Indeed, Tamburini et al. demonstrated that LEC proliferation coupled with antigen capture leads to prolonged antigen expression within LECs, increasing IFNγ and IL-2 production, and enhancing protection against infections. While the delivery of MHCI and MHCII-restricted antigens resulted in dysfunctional activation of CD8^+^ and CD4^+^ T cell responses, it is unclear what role the contribution of LEC antigen-presentation played.

### Targeting Lymphatic Vessels for Immunomodulation

With a key role in immunomodulation, lymphatic vessels themselves have emerged as an attractive therapeutic target. One of the key targets for potentially targeting lymphatic vessels themselves is targeting lymphatic-specific receptors, such as VEGFR-3 or Lyve-1. VEGFR-3 is part of the family of vascular endothelial growth factor receptors and is mainly appreciated for promoting lymphangiogenesis (Witmer et al., 2001). Lyve-1, or lymphatic vessel endothelial hyaluronan receptor 1, is a common, integral membrane protein used for identifying LECs, but can also be found in liver blood sinusoids, embryonic blood vessels, and certain subsets of macrophages (Mouta Carreira et al., 2001; Gordon et al., 2008; Brezovakova and Jadhav, 2020). Guo and colleagues targeted LECs *via* Lyve-1 *in vitro*, by using Lyve-1-binding PEG to ultrasmall superparamagnetic iron oxide nanoparticles as a potential MRI contrast agent (Guo et al., 2013). Dashkevich et al. were able to leverage this VEGFR-3 targeting to prevent cardiac allograft rejection. They demonstrated that inhibition of VEGFR-3 led to reduced CCL21 production (Dashkevich et al., 2016). They found that this reduced CCL21 production did not affect lymphangiogenesis within the graft but did result in lower concentrations of CD8^+^ T cells within the graft. In an additional experiment, they demonstrated that treatment with a neutralizing monoclonal VEGFR-3 antibody reduced arteriosclerosis, the number of activated lymphatic vessels expressing VEGFR-3 and CCL21, and graft-infiltrating CD4^+^ T cells in chronically rejecting mouse cardiac allografts. Blocking the interaction between VEGFR-3 on lymphatic vessels and VEGF-C produced by tumors can be a form of tumor suppression through prevention of tumor lymphangiogenesis, which contributes to metastasis and tumor growth (Su et al., 2007). Maisel et al. have demonstrated that VEGFR-3 activation in allergic responses can initiate acute inflammatory response and regulate the adaptive immune response. They found that blocking VEGFR-3 leads to less inflammation, reduced innate and T-cell numbers, and reduced inflammatory chemokine levels initially. However, VEGFR-3 blocking also significantly enhanced memory T cell responses to allergens, suggesting that targeting lymphangiogenesis in inflammatory conditions may be a double-edged sword (Maisel et al., 2021). In cancer, studies have demonstrated that treatment with VEGF-C, the ligand for VEGFR-3, can improve immunotherapy treatments in melanoma (Yeh et al., 2017). Fankhauser et al. were able to demonstrate that while VEGF-C can promote angiogenesis, and subsequent metastasis, it also potentiates immunotherapy by attracting naïve T cells (Fankhauser et al., 2017). The group also demonstrated that positive response to immunotherapy in patients can be correlated with expression of lymphatic-related markers within the tumors, suggesting that lymphatics have a role in enhancing tumor immunotherapies. Huggenberger et al. have also show demonstrated that stimulation of VEGFR-3 with VEGF-C along with antiangiogenic treatment could be used to treat chronic inflammation within the skin. However, other studies by Wang et al. and Krebs et al. have highlighted that inhibiting VEGFR-3 activation can result in deterioration of inflammation in acute and chronic colitis, and immune responses to obliterative airway disease (Krebs et al., 2012; Wang et al., 2016).

## Outlook

Lymphatic vessels have become a tissue of interest in therapeutic design in large part for their role in transport as well as immunomodulation. Lymphatic vessels can effectively transport immunomodulatory therapies after non-invasive oral or intradermal/subcutaneous delivery, which in turn can improve the efficacy of immunomodulatory therapies that need to reach lymph nodes for improved therapeutic efficacy. While we have scratched the surface of how to target lymphatic transport, we still have an incomplete understanding of lymphatic functions, and how to target specific immune cell populations after transport to lymph nodes [more on targeting lymph node components can be found in this excellent review (Schudel et al., 2019)]. Continued exploration of how targeting lymphatic functions can serve as a therapeutic target will lead to new discoveries and advances that can further enhance immune modulatory therapeutics and lead to new treatments.

## References

[B1] Aspelund A. Robciuc M. R. Karaman S. Makinen T. Alitalo K. (2016). Lymphatic System in Cardiovascular Medicine. Circ. Res. 118 (3), 515–530. 10.1161/circresaha.115.306544 26846644

[B2] Baek J. S. Cho C. W. (2017). Surface Modification of Solid Lipid Nanoparticles for Oral Delivery of Curcumin: Improvement of Bioavailability through Enhanced Cellular Uptake, and Lymphatic Uptake. Eur. J. Pharm. Biopharm. 117, 132–140. 10.1016/j.ejpb.2017.04.013 28412471

[B3] Baluk P. Fuxe J. Hashizume H. Romano T. Lashnits E. Butz S. (2007). Functionally Specialized Junctions between Endothelial Cells of Lymphatic Vessels. J. Exp. Med. 204 (10), 2349–2362. 10.1084/jem.20062596 17846148 PMC2118470

[B4] Breslin J. W. Yang Y. Scallan J. P. Sweat R. S. Adderley S. P. Murfee W. L. (2018). Lymphatic Vessel Network Structure and Physiology. Compr. Physiol. 9 (1), 207–299. 10.1002/cphy.c180015 30549020 PMC6459625

[B5] Brezovakova V. Jadhav S. (2020). Identification of Lyve-1 Positive Macrophages as Resident Cells in Meninges of Rats. J. Comp. Neurol. 528 (12), 2021–2032. 10.1002/cne.24870 32003471

[B6] Casley-Smith J. R. (1985). The Importance of the Lymphatic System. Angiology 36 (3), 201–202. 10.1177/000331978503600310 4025932

[B7] Chaudhary S. Garg T. Murthy R. S. Rath G. Goyal A. K. (2014). Recent Approaches of Lipid-Based Delivery System for Lymphatic Targeting via Oral Route. J. Drug Target 22 (10), 871–882. 10.3109/1061186x.2014.950664 25148607

[B8] Chen Y. De Koker S. De Geest B. G. (2020). Engineering Strategies for Lymph Node Targeted Immune Activation. Acc. Chem. Res. 53 (10), 2055–2067. 10.1021/acs.accounts.0c00260 32910636

[B9] Comerford I. Harata-Lee Y. Bunting M. D. Gregor C. Kara E. E. McColl S. R. (2013). A Myriad of Functions and Complex Regulation of the CCR7/CCL19/CCL21 Chemokine axis in the Adaptive Immune System. Cytokine Growth Factor Rev. 24 (3), 269–283. 10.1016/j.cytogfr.2013.03.001 23587803

[B10] Cooper A. D. (1997). Hepatic Uptake of Chylomicron Remnants. J. Lipid Res. 38 (11), 2173–2192. 10.1016/s0022-2275(20)34932-4 9392416

[B11] Cromer W. E. Zawieja S. D. Tharakan B. Childs E. W. Newell M. K. Zawieja D. C. (2014). The Effects of Inflammatory Cytokines on Lymphatic Endothelial Barrier Function. Angiogenesis 17 (2), 395–406. 10.1007/s10456-013-9393-2 24141404 PMC4314095

[B12] Dahan A. Zimmermann E. M. Ben-Shabat S. (2014). Modern Prodrug Design for Targeted Oral Drug Delivery. Molecules 19 (10), 16489–16505. 10.3390/molecules191016489 25317578 PMC6271014

[B13] Dashkevich A. Raissadati A. Syrjälä S. O. Zarkada G. Keränen M. A. Tuuminen R. (2016). Ischemia-Reperfusion Injury Enhances Lymphatic Endothelial VEGFR3 and Rejection in Cardiac Allografts. Am. J. Transpl. 16 (4), 1160–1172. 10.1111/ajt.13564 26689983

[B14] De Koker S. Cui J. Vanparijs N. Albertazzi L. Grooten J. Caruso F. (2016). Engineering Polymer Hydrogel Nanoparticles for Lymph Node-Targeted Delivery. Angew. Chem. Int. Ed. Engl. 55 (4), 1334–1339. 10.1002/anie.201508626 26666207

[B15] Dejana E. Orsenigo F. Molendini C. Baluk P. McDonald D. M. (2009). Organization and Signaling of Endothelial Cell-To-Cell Junctions in Various Regions of the Blood and Lymphatic Vascular Trees. Cell. Tissue Res. 335 (1), 17–25. 10.1007/s00441-008-0694-5 18855014 PMC4422058

[B16] Fankhauser M. Broggi M. A. S. Potin L. Bordry N. Jeanbart L. Lund A. W. (2017). Tumor Lymphangiogenesis Promotes T Cell Infiltration and Potentiates Immunotherapy in Melanoma. Sci. Transl. Med. 9 (407). 10.1126/scitranslmed.aal4712 28904226

[B17] Farnsworth R. H. Karnezis T. Maciburko S. J. Mueller S. N. Stacker S. A. (2019). The Interplay between Lymphatic Vessels and Chemokines. Front. Immunol. 10, 518. 10.3389/fimmu.2019.00518 31105685 PMC6499173

[B18] Garnier L. Gkountidi A. O. Hugues S. (2019). Tumor-Associated Lymphatic Vessel Features and Immunomodulatory Functions. Front. Immunol. 10, 720. 10.3389/fimmu.2019.00720 31024552 PMC6465591

[B19] Geng X. Cha B. Mahamud M. R. Lim K. C. Silasi-Mansat R. Uddin M. K. M. (2016). Multiple Mouse Models of Primary Lymphedema Exhibit Distinct Defects in Lymphovenous Valve Development. Dev. Biol. 409 (1), 218–233. 10.1016/j.ydbio.2015.10.022 26542011 PMC4688075

[B20] Gordon E. J. Gale N. W. Harvey N. L. (2008). Expression of the Hyaluronan Receptor LYVE-1 Is Not Restricted to the Lymphatic Vasculature; LYVE-1 Is Also Expressed on Embryonic Blood Vessels. Dev. Dyn. 237 (7), 1901–1909. 10.1002/dvdy.21605 18570254

[B21] Gorlino C. V. Ranocchia R. P. Harman M. F. García I. A. Crespo M. I. Morón G. (2014). Neutrophils Exhibit Differential Requirements for Homing Molecules in Their Lymphatic and Blood Trafficking into Draining Lymph Nodes. J. Immunol. 193 (4), 1966–1974. 10.4049/jimmunol.1301791 25015824

[B22] Gracia G. Cao E. Feeney O. M. Johnston A. P. R. Porter C. J. H. Trevaskis N. L. (2020). High-Density Lipoprotein Composition Influences Lymphatic Transport after Subcutaneous Administration. Mol. Pharm. 17 (8), 2938–2951. 10.1021/acs.molpharmaceut.0c00348 32543863

[B23] Guo Q. Liu Y. Xu K. Ren K. Sun W. (2013). Mouse Lymphatic Endothelial Cell Targeted Probes: Anti-LYVE-1 Antibody-Based Magnetic Nanoparticles. Int. J. Nanomedicine 8, 2273–2284. 10.2147/IJN.S45817 23818783 PMC3693816

[B24] Gupta A. Moore J. A. (2018). Lymphedema. JAMA Oncol. 4 (5), 755. 10.1001/jamaoncol.2017.5553 29494737

[B25] Gutjahr A. Phelip C. Coolen A. L. Monge C. Boisgard A. S. Paul S. (2016). Biodegradable Polymeric Nanoparticles-Based Vaccine Adjuvants for Lymph Nodes Targeting. Vaccines (Basel) 4 (4), 34. 10.3390/vaccines4040034 PMC5192354 27754314

[B26] Haig D. M. Hopkins J. Miller H. R. (1999). Local Immune Responses in Afferent and Efferent Lymph. Immunology 96 (2), 155–163. 10.1046/j.1365-2567.1999.00681.x 10233690 PMC2326739

[B27] Hampton H. R. Chtanova T. (2019). Lymphatic Migration of Immune Cells. Front. Immunol. 10, 1168. 10.3389/fimmu.2019.01168 31191539 PMC6546724

[B28] Hematti H. Mehran R. J. (2011). Anatomy of the Thoracic Duct. Thorac. Surg. Clin. 21 (2), 229ix–ix. 10.1016/j.thorsurg.2011.01.002 21477773

[B29] Ikomi F. Hunt J. Hanna G. Schmid-Schönbein G. W. (1996). Interstitial Fluid, Plasma Protein, Colloid, and Leukocyte Uptake into Initial Lymphatics. J. Appl. Physiol. (1985) 81 (5), 2060–2067. 10.1152/jappl.1996.81.5.2060 8941530

[B30] Ivanov S. Scallan J. P. Kim K. W. Werth K. Johnson M. W. Saunders B. T. (2016). CCR7 and IRF4-dependent Dendritic Cells Regulate Lymphatic Collecting Vessel Permeability. J. Clin. Invest. 126 (4), 1581–1591. 10.1172/jci84518 26999610 PMC4811132

[B31] Kakei Y. Akashi M. Shigeta T. Hasegawa T. Komori T. (2014). Alteration of Cell-Cell Junctions in Cultured Human Lymphatic Endothelial Cells with Inflammatory Cytokine Stimulation. Lymphat. Res. Biol. 12, 136–143. 10.1089/lrb.2013.0035 25166264 PMC4170982

[B32] Kaminskas L. M. Kota J. McLeod V. M. Kelly B. D. Karellas P. Porter C. J. (2009). PEGylation of Polylysine Dendrimers Improves Absorption and Lymphatic Targeting Following SC Administration in Rats. J. Control Release 140 (2), 108–116. 10.1016/j.jconrel.2009.08.005 19686787

[B33] Kastenmüller W. Torabi-Parizi P. Subramanian N. Lämmermann T. Germain R. N. (2012). A Spatially-Organized Multicellular Innate Immune Response in Lymph Nodes Limits Systemic Pathogen Spread. Cell. 150 (6), 1235–1248. 10.1016/j.cell.2012.07.021 22980983 PMC3514884

[B34] Kim K. S. Suzuki K. Cho H. Youn Y. S. Bae Y. H. (2018). Oral Nanoparticles Exhibit Specific High-Efficiency Intestinal Uptake and Lymphatic Transport. ACS Nano 12 (9), 8893–8900. 10.1021/acsnano.8b04315 30088412 PMC6377080

[B35] Kim K. S. Youn Y. S. Bae Y. H. (2019). Immune-triggered Cancer Treatment by Intestinal Lymphatic Delivery of Docetaxel-Loaded Nanoparticle. J. Control Release 311-312, 85–95. 10.1016/j.jconrel.2019.08.027 31461664

[B36] Kim J. Archer P. A. Thomas S. N. (2021). Innovations in Lymph Node Targeting Nanocarriers. Semin. Immunol. 56, 101534. 10.1016/j.smim.2021.101534 34836772 PMC8792237

[B37] Kobayashi H. Kawamoto S. Bernardo M. Brechbiel M. W. Knopp M. V. Choyke P. L. (2006). Delivery of Gadolinium-Labeled Nanoparticles to the Sentinel Lymph Node: Comparison of the Sentinel Node Visualization and Estimations of Intra-nodal Gadolinium Concentration by the Magnetic Resonance Imaging. J. Control Release 111 (3), 343–351. 10.1016/j.jconrel.2005.12.019 16490277

[B38] Kochappan R. Cao E. Han S. Hu L. Quach T. Senyschyn D. (2021). Targeted Delivery of Mycophenolic Acid to the Mesenteric Lymph Node Using a Triglyceride Mimetic Prodrug Approach Enhances Gut-specific Immunomodulation in Mice. J. Control Release 332, 636–651. 10.1016/j.jconrel.2021.02.008 33609620

[B39] Krebs R. Tikkanen J. M. Ropponen J. O. Jeltsch M. Jokinen J. J. Ylä-Herttuala S. (2012). Critical Role of VEGF-C/VEGFR-3 Signaling in Innate and Adaptive Immune Responses in Experimental Obliterative Bronchiolitis. Am. J. Pathol. 181 (5), 1607–1620. 10.1016/j.ajpath.2012.07.021 22959907

[B40] Kriehuber E. Breiteneder-Geleff S. Groeger M. Soleiman A. Schoppmann S. F. Stingl G. (2001). Isolation and Characterization of Dermal Lymphatic and Blood Endothelial Cells Reveal Stable and Functionally Specialized Cell Lineages. J. Exp. Med. 194 (6), 797–808. 10.1084/jem.194.6.797 11560995 PMC2195953

[B41] Lee G. Han S. Inocencio I. Cao E. Hong J. Phillips A. R. J. (2019). Lymphatic Uptake of Liposomes after Intraperitoneal Administration Primarily Occurs via the Diaphragmatic Lymphatics and Is Dependent on Liposome Surface Properties. Mol. Pharm. 16 (12), 4987–4999. 10.1021/acs.molpharmaceut.9b00855 31625752

[B42] Lee G. Han S. Lu Z. Hong J. Phillips A. R. J. Windsor J. A. (2021). Intestinal Delivery in a Long-Chain Fatty Acid Formulation Enables Lymphatic Transport and Systemic Exposure of Orlistat. Int. J. Pharm. 596, 120247. 10.1016/j.ijpharm.2021.120247 33486039

[B43] Lucas E. D. Tamburini B. A. J. (2019). Lymph Node Lymphatic Endothelial Cell Expansion and Contraction and the Programming of the Immune Response. Front. Immunol. 10, 36. 10.3389/fimmu.2019.00036 30740101 PMC6357284

[B44] Lukacs-Kornek V. Malhotra D. Fletcher A. L. Acton S. E. Elpek K. G. Tayalia P. (2011). Regulated Release of Nitric Oxide by Nonhematopoietic Stroma Controls Expansion of the Activated T Cell Pool in Lymph Nodes. Nat. Immunol. 12 (11), 1096–1104. 10.1038/ni.2112 21926986 PMC3457791

[B45] Maisel K. Hrusch C. L. Medellin J. E. G. Potin L. Chapel D. B. Nurmi H. (2021). Pro-lymphangiogenic VEGFR-3 Signaling Modulates Memory T Cell Responses in Allergic Airway Inflammation. Mucosal Immunol. 14 (1), 144–151. 10.1038/s41385-020-0308-4 32518367 PMC7725864

[B46] Mao Y. Feng S. Li S. Zhao Q. Di D. Liu Y. (2019). Chylomicron-pretended Nano-Bio Self-Assembling Vehicle to Promote Lymphatic Transport and GALTs Target of Oral Drugs. Biomaterials 188, 173–186. 10.1016/j.biomaterials.2018.10.012 30359884

[B47] McCright J. Skeen C. Yarmovsky J. Maisel K. (2020). Dense Poly(ethylene Glycol) Coatings Maximize Nanoparticle Transport across Lymphatic Endothelial Cells. bioRxiv 2020, 232249. 10.1101/2020.08.01.232249

[B48] McCright J. Skeen C. Yarmovsky J. Maisel K. (2022). Nanoparticles with Dense Poly(ethylene Glycol) Coatings with Near Neutral Charge Are Maximally Transported across Lymphatics and to the Lymph Nodes. Acta Biomater. 10.1016/j.actbio.2022.03.054 PMC9133124 35381399

[B49] Miteva D. O. Rutkowski J. M. Dixon J. B. Kilarski W. Shields J. D. Swartz M. A. (2010). Transmural Flow Modulates Cell and Fluid Transport Functions of Lymphatic Endothelium. Circ. Res. 106 (5), 920–931. 10.1161/circresaha.109.207274 20133901 PMC10994404

[B50] Moghimi S. M. Hawley A. E. Christy N. M. Gray T. Illum L. Davis S. S. (1994). Surface Engineered Nanospheres with Enhanced Drainage into Lymphatics and Uptake by Macrophages of the Regional Lymph Nodes. FEBS Lett. 344 (1), 25–30. 10.1016/0014-5793(94)00351-3 8181558

[B51] Mouta Carreira C. Nasser S. M. di Tomaso E. Padera T. P. Boucher Y. Tomarev S. I. (2001). LYVE-1 Is Not Restricted to the Lymph Vessels: Expression in Normal Liver Blood Sinusoids and Down-Regulation in Human Liver Cancer and Cirrhosis. Cancer Res. 61 (22), 8079–8084. 11719431

[B52] Nakamura T. Kawai M. Sato Y. Maeki M. Tokeshi M. Harashima H. (2020). The Effect of Size and Charge of Lipid Nanoparticles Prepared by Microfluidic Mixing on Their Lymph Node Transitivity and Distribution. Mol. Pharm. 17 (3), 944–953. 10.1021/acs.molpharmaceut.9b01182 31990567

[B53] Nishimoto Y. Nagashima S. Nakajima K. Ohira T. Sato T. Izawa T. (2020). Carboxyl-, Sulfonyl-, and Phosphate-Terminal Dendrimers as a Nanoplatform with Lymph Node Targeting. Int. J. Pharm. 576, 119021. 10.1016/j.ijpharm.2020.119021 31917298

[B54] Nykänen A. I. Sandelin H. Krebs R. Keränen M. A. I. Tuuminen R. Kärpänen T. (2010). Targeting Lymphatic Vessel Activation and CCL21 Production by Vascular Endothelial Growth Factor Receptor-3 Inhibition Has Novel Immunomodulatory and Antiarteriosclerotic Effects in Cardiac Allografts. Circulation 121(12), 1413–1422. doi:10.1161/CIRCULATIONAHA.109.910703 20231530

[B55] Trevaskis N. L. Hu L. Caliph S. M. Han S. Porter C. J., (2015). The Mesenteric Lymph Duct Cannulated Rat Model: Application to the Assessment of Intestinal Lymphatic Drug Transport. J. Vis. Exp. 97, e52389. doi:10.3791/52389 PMC4401200 25866901

[B56] Pflicke H. Sixt M. (2009). Preformed Portals Facilitate Dendritic Cell Entry into Afferent Lymphatic Vessels. J. Exp. Med. 206 (13), 2925–2935. 10.1084/jem.20091739 19995949 PMC2806476

[B57] Rahbar E. Akl T. Coté G. L. Moore J. E. Zawieja D. C. (2014). Lymph Transport in Rat Mesenteric Lymphatics Experiencing Edemagenic Stress. Microcirculation 21 (5), 359–367. 10.1111/micc.12112 24397756 PMC4174575

[B58] Rao D. A. Forrest M. L. Alani A. W. Kwon G. S. Robinson J. R. (2010). Biodegradable PLGA Based Nanoparticles for Sustained Regional Lymphatic Drug Delivery. J. Pharm. Sci. 99 (4), 2018–2031. 10.1002/jps.21970 19902520 PMC5178132

[B59] Reddy S. T. van der Vlies A. J. Simeoni E. Angeli V. Randolph G. J. O'Neil C. P. (2007). Exploiting Lymphatic Transport and Complement Activation in Nanoparticle Vaccines. Nat. Biotechnol. 25 (10), 1159–1164. 10.1038/nbt1332 17873867

[B60] Rigby D. A. Ferguson D. J. Johnson L. A. Jackson D. G. (2015). Neutrophils Rapidly Transit Inflamed Lymphatic Vessel Endothelium via Integrin-dependent Proteolysis and Lipoxin-Induced Junctional Retraction. J. Leukoc. Biol. 98 (6), 897–912. 10.1189/jlb.1HI0415-149R 26216937

[B61] Rockson S. G. (2001). Lymphedema. Am. J. Med. 110 (4), 288–295. 10.1016/s0002-9343(00)00727-0 11239847

[B62] Rohner N. A. Thomas S. N. (2017). Flexible Macromolecule versus Rigid Particle Retention in the Injected Skin and Accumulation in Draining Lymph Nodes Are Differentially Influenced by Hydrodynamic Size. ACS Biomaterials Sci. Eng. 3 (2), 153–159. 10.1021/acsbiomaterials.6b00438 PMC5990040 29888321

[B64] Sabine A. Bovay E. Demir C. S. Kimura W. Jaquet M. Agalarov Y. (2015). FOXC2 and Fluid Shear Stress Stabilize Postnatal Lymphatic Vasculature. J. Clin. Invest. 125 (10), 3861–3877. 10.1172/jci80454 26389677 PMC4607114

[B65] Sallusto F. Lenig D. Förster R. Lipp M. Lanzavecchia A. (1999). Two Subsets of Memory T Lymphocytes with Distinct Homing Potentials and Effector Functions. Nature 401 (6754), 708–712. 10.1038/44385 10537110

[B66] Scallan J. Huxley V. H. Korthuis R. J. (2010). “Integrated Systems Physiology: from Molecule to Function to Disease,” in Capillary Fluid Exchange: Regulation, Functions, and Pathology (Morgan & Claypool Life Sciences). 10.4199/C00006ED1V01Y201002ISP003 21452435

[B67] Schudel A. Chapman A. P. Yau M. K. Higginson C. J. Francis D. M. Manspeaker M. P. (2020). Programmable Multistage Drug Delivery to Lymph Nodes. Nat. Nanotechnol. 15 (6), 491–499. 10.1038/s41565-020-0679-4 32523099 PMC7305972

[B68] Schudel A. Francis D. M. Thomas S. N. (2019). Material Design for Lymph Node Drug Delivery. Nat. Rev. Mater 4 (6), 415–428. 10.1038/s41578-019-0110-7 32523780 PMC7286627

[B69] Schulte-Merker S. Sabine A. Petrova T. V. (2011). Lymphatic Vascular Morphogenesis in Development, Physiology, and Disease. J. Cell. Biol. 193 (4), 607–618. 10.1083/jcb.201012094 21576390 PMC3166860

[B70] Schwager S. Detmar M. (2019). Inflammation and Lymphatic Function. Front. Immunol. 10, 308. 10.3389/fimmu.2019.00308 30863410 PMC6399417

[B71] Schwartz L. H. Gandras E. J. Colangelo S. M. Ercolani M. C. Panicek D. M. (1999). Prevalence and Importance of Small Hepatic Lesions Found at CT in Patients with Cancer. Radiology 210 (1), 71–74. 10.1148/radiology.210.1.r99ja0371 9885589

[B72] Srinivasan S. Vannberg F. O. Dixon J. B. (2016). Lymphatic Transport of Exosomes as a Rapid Route of Information Dissemination to the Lymph Node. Sci. Rep. 6 (1), 24436. 10.1038/srep24436 27087234 PMC4834495

[B73] Su J. L. Yen C. J. Chen P. S. Chuang S. E. Hong C. C. Kuo I. H. (2007). The Role of the VEGF-C/VEGFR-3 axis in Cancer Progression. Br. J. Cancer 96 (4), 541–545. 10.1038/sj.bjc.6603487 17164762 PMC2360045

[B74] Suzuki K. Kim K. S. Bae Y. H. (2019). Long-term Oral Administration of Exendin-4 to Control Type 2 Diabetes in a Rat Model. J. Control Release 294, 259–267. 10.1016/j.jconrel.2018.12.028 30572033 PMC6369909

[B75] Swartz M. A. (2001). The Physiology of the Lymphatic System. Adv. Drug Deliv. Rev. 50 (1-2), 3–20. 10.1016/s0169-409x(01)00150-8 11489331

[B76] Takeda A. Hollmén M. Dermadi D. Pan J. Brulois K. F. Kaukonen R. (2019). Single-Cell Survey of Human Lymphatics Unveils Marked Endothelial Cell Heterogeneity and Mechanisms of Homing for Neutrophils. Immunity 51 (3), 561e5–e5. 10.1016/j.immuni.2019.06.027 31402260

[B77] Tamburini B. A. Burchill M. A. Kedl R. M. (2014). Antigen Capture and Archiving by Lymphatic Endothelial Cells Following Vaccination or Viral Infection. Nat. Commun. 5, 3989. 10.1038/ncomms4989 24905362 PMC4073648

[B78] Tecchio C. Micheletti A. Cassatella M. A. (2014). Neutrophil-derived Cytokines: Facts beyond Expression. Front. Immunol. 5, 508. 10.3389/fimmu.2014.00508 25374568 PMC4204637

[B79] Trevaskis N. L. Kaminskas L. M. Porter C. J. (2015). From Sewer to Saviour - Targeting the Lymphatic System to Promote Drug Exposure and Activity. Nat. Rev. Drug Discov. 14 (11), 781–803. 10.1038/nrd4608 26471369

[B80] Triacca V. Güç E. Kilarski W. W. Pisano M. Swartz M. A. (2017). Transcellular Pathways in Lymphatic Endothelial Cells Regulate Changes in Solute Transport by Fluid Stress. Circ. Res. 120 (9), 1440–1452. 10.1161/CIRCRESAHA.116.309828 28130294

[B81] Trzewik J. Mallipattu S. K. Artmann G. M. Delano F. A. Schmid-Schönbein G. W. (2001). Evidence for a Second Valve System in Lymphatics: Endothelial Microvalves. Faseb J. 15 (10), 1711–1717. 10.1096/fj.01-0067com 11481218

[B82] Varypataki E. M. Silva A. L. Barnier-Quer C. Collin N. Ossendorp F. Jiskoot W. (2016). Synthetic Long Peptide-Based Vaccine Formulations for Induction of Cell Mediated Immunity: A Comparative Study of Cationic Liposomes and PLGA Nanoparticles. J. Control Release 226, 98–106. 10.1016/j.jconrel.2016.02.018 26876760

[B83] Velanovich V. Szymanski W. (1999). Quality of Life of Breast Cancer Patients with Lymphedema. Am. J. Surg. 177 (3), 184–188. discussion 188 (1999). 10.1016/s0002-9610(99)00008-2 10219851

[B84] Vigl B. Aebischer D. Nitschké M. Iolyeva M. Röthlin T. Antsiferova O. (2011). Tissue Inflammation Modulates Gene Expression of Lymphatic Endothelial Cells and Dendritic Cell Migration in a Stimulus-dependent Manner. Blood 118 (1), 205–215. 10.1182/blood-2010-12-326447 21596851

[B85] Wang X. L. Zhao J. Qin L. Cao J. L. (2016). VEGFR-3 Blocking Deteriorates Inflammation with Impaired Lymphatic Function and Different Changes in Lymphatic Vessels in Acute and Chronic Colitis. Am. J. Transl. Res. 8 (2), 827–841. 27158372 PMC4846929

[B86] Witmer A. N. van Blijswijk B. C. Dai J. Hofman P. Partanen T. A. Vrensen G. F. (2001). VEGFR-3 in Adult Angiogenesis. J. Pathol. 195 (4), 490–497. 10.1002/path.969 11745682

[B87] Yao L. C. Baluk P. Srinivasan R. S. Oliver G. McDonald D. M. (2012). Plasticity of Button-like Junctions in the Endothelium of Airway Lymphatics in Development and Inflammation. Am. J. Pathol. 180 (6), 2561–2575. 10.1016/j.ajpath.2012.02.019 22538088 PMC3378913

[B88] Yeh Y. W. Cheng C. C. Yang S. T. Tseng C. F. Chang T. Y. Tsai S. Y. (2017). Targeting the VEGF-C/VEGFR3 axis Suppresses Slug-Mediated Cancer Metastasis and Stemness via Inhibition of KRAS/YAP1 Signaling. Oncotarget 8 (3), 5603–5618. 10.18632/oncotarget.13629 27901498 PMC5354933

[B89] Zeng Q. Jiang H. Wang T. Zhang Z. Gong T. Sun X. (2015). Cationic Micelle Delivery of Trp2 Peptide for Efficient Lymphatic Draining and Enhanced Cytotoxic T-Lymphocyte Responses. J. Control Release 200, 1–12. 10.1016/j.jconrel.2014.12.024 25540903

[B90] Zhang F. Zarkada G. Yi S. Eichmann A. (2020). Lymphatic Endothelial Cell Junctions: Molecular Regulation in Physiology and Diseases. Front. Physiol. 11, 509. 10.3389/fphys.2020.00509 32547411 PMC7274196

[B91] Zhuang Y. Ma Y. Wang C. Hai L. Yan C. Zhang Y. (2012). PEGylated Cationic Liposomes Robustly Augment Vaccine-Induced Immune Responses: Role of Lymphatic Trafficking and Biodistribution. J. Control Release 159 (1), 135–142. 10.1016/j.jconrel.2011.12.017 22226776

